# Effects of Shading on Metabolism and Grain Yield of Irrigated Rice During Crop Development

**DOI:** 10.3390/plants14162491

**Published:** 2025-08-11

**Authors:** Stefânia Nunes Pires, Fernanda Reolon de Souza, Bruna Evelyn Paschoal Silva, Natan da Silva Fagundes, Simone Ribeiro Lucho, Luis Antonio de Avila, Sidnei Deuner

**Affiliations:** 1 Department of Botany, Federal University of Pelotas, Pelotas 96010-610, RS, Brazil; stefanianunespires@gmail.com (S.N.P.); brunabiologia89@hotmail.com (B.E.P.S.); natanfagundes@gmail.com (N.d.S.F.); simonibelmonte@gmail.com (S.R.L.); sdeuner@yahoo.com.br (S.D.); 2 Department of Plant and Soil Sciences, Mississippi State University, Starkville, MS 37962, USA; luis.avila@msstate.edu

**Keywords:** *Oryza sativa* L., shading, metabolism, productivity, enzymes activities

## Abstract

Rice (*Oryza sativa* L.) plays a pivotal role in the Brazilian economy, serving as a staple food for more than half of the world’s population and thereby contributing to global food security. Projections of future climate change scenarios indicate an increase in extreme weather events. Among climate variables that impact the development and productivity of irrigated rice, solar radiation is one of the most important in defining productive potential. Understanding the risks imposed on agricultural production by the occurrence of days with reduced luminosity availability is crucial for guiding adequate responses that mitigate the negative impacts of climate variability. Therefore, this study aimed to investigate the effect of shade on the metabolism and productivity of irrigated rice plants, with a specific focus on the synthesis of photosynthetic pigments, carbohydrate accumulation, invertase activity, and the nutritional status and grain yield of rice. For this, the study was conducted on the field rice cultivars IRGA 424 RI, BRS PAMPA, and BRS PAMPEIRA, which were subjected to 35% shading using black nylon netting installed when the plants reached the reproductive stage (R0). The restriction was maintained until the R4 stage, and later, from the R4 stage until the R9 stage. After the imposition of treatments, evaluations took place at the phenological stages R2, R4, R6, and R8. In shaded plants, a higher content of photosynthetic pigments and a lower accumulation of carbohydrates were observed, which was reflected in an increase in the activity of invertase enzymes. These conditions were able to potentiate effects on the nutritional status of the plants, in addition to reducing productivity and 1000-grain weight and increasing spikelet sterility, due to changes in the source–sink relationship, with effects more pronounced in cultivars BRS PAMPA and BRS PAMPEIRA during the R4–R9 period.

## 1. Introduction

Food availability is among the priorities of countries due to population growth. In this context, rice stands out as the staple food for more than half of the world’s population, playing a crucial role in ensuring food security. With the growing demand, increasing yield per area is a strategy that can guarantee an improvement in grain production [1,2,3]. Brazil is the largest rice producer outside of Asia, with an estimated production of 11 million metric tons (MMT) for the 2024/2025 season [4]. In southern Brazil, irrigated rice is primarily cultivated between latitudes 28° and 33° S, with the state of Rio Grande do Sul accounting for approximately 69% of national production [5].

Among the rice cultivars, IRGA 424 RI leads with 54.47% of the total area cultivated [5]. Other important varieties in Rio Grande do Sul include BRS Pampa and BRS Pampeira. BRS Pampa is known for its high yield potential and excellent grain quality. In contrast, BRS Pampeira stands out for its adaptability to diverse regions of the state, including areas with lower temperatures, as well as its strong resistance to diseases.

During their development, crops face several biotic and abiotic stress factors. Among the abiotic stresses, extreme temperatures and environmental fluctuations are the most recurrent [6,7,8]. As an energy source, light is crucial for the photosynthetic rate and carbon assimilation and accumulation, in addition to its regulatory roles in growth and productivity [9,10]. Plants are efficient at converting sunlight into sugars through the process of photosynthetic carbon fixation, a process that occurs in their tissues [11]. Decreased sunlight to plants due to high cloud intensity is a significant cause of reduced plant capacity to produce carbohydrates, limiting the growth rate and stability under field conditions [12,13].

Cloud shading in rice plants negatively impacts metabolic processes, leading to a decreased photosynthetic rate and carbohydrate synthesis, as well as reduced nitrogen fixation, and consequently, lower productivity, especially during the reproductive period [14]. Additionally, shading decreases the rate of electron transport from photosystem II to photosystem I, which results in lower ATP production and reduced Rubisco activity. Excessive shading can impair light-use efficiency by altering the proportions of chlorophyll *a*, chlorophyll *b*, and total chlorophyll [15,16]. Shading also reduces non-structural carbohydrates, such as soluble sugars, starch, and sucrose, which provide energy and contribute to the accumulation of structural carbohydrates [17]. After sugar synthesis in plants, only some sugars are transported over long distances, like sucrose. Upon reaching sink tissues, sucrose can follow different pathways, influencing sink strength and carbon flux [18]. Sucrose can be unloaded from the phloem into the apoplast via transporters or hydrolyzed by invertase enzymes to form glucose and fructose [19].

Nitrogen concentrations in plant tissues vary significantly depending on the availability of resources, including light and nutrients. Nitrogen is essential for photosynthesis and facilitates carbohydrate accumulation in stems and leaf sheaths during panicle initiation, as well as in grains during grain filling. Generally, nitrogen levels decrease in plants under low-light conditions [20,21]. Sun et al. [22] reported that a 40% reduction in natural light during the R3 stage in rice hybrids results in lower dry matter accumulation and altered redistribution of photosynthetic products.

Furthermore, low light during flowering is associated with increased spikelet sterility, which correlates with a decrease in carbohydrate content and reduced protein synthesis [23]. Identifying metabolic pathways and compensation mechanisms is vital for understanding how shade stress impacts crop productivity. Therefore, the objective of this study was to investigate the impact of shade on the metabolism and productivity of irrigated rice plants, with a specific focus on the synthesis of photosynthetic pigments, carbohydrate accumulation, invertase activity, and the nutritional status and grain yield of rice.

## 2. Materials and Methods

### 2.1. Plant Material and Growth Conditions

**Figure 1:**
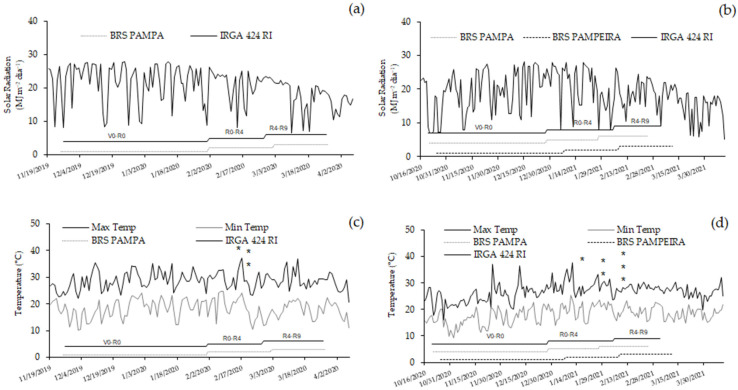
Maximum and minimum temperatures (°C) during the crop development cycle for the 2019/20 (**a**) and 2020/21 (**b**) harvests. Asterisks represent the beginning of flowering of the cultivars: * BRS PAMPA, ** IRGA 424 RI, and *** BRS PAMPEIRA. Solar radiation (MJ m^−2^) during the crop development cycle for the 2019/20 (**c**) and 2020/21 (**d**) harvests. Font: EMBRAPA [22].

This experiment was conducted in the field at the Centro Agropecuário da Palma, located in the municipality of Capão do Leão, RS, Brazil (31°48′6″ S, 52°31′1″ W), an area belonging to the Federal University of Pelotas (UFPel), during the 2019/20 and 2020/21 agricultural years. Three irrigated rice cultivars were used, one with an early cycle, BRS Pampa (113 to 123 days), and two with a medium cycle, BRS Pampeira (133 days) and IRGA 424 RI (133 days). For each cultivar, plots measuring 5.0 m in length and 1.53 m in width were prepared in a randomized block design with four replications. Sowing in the 2019/20 harvest took place in the first fortnight of November, and the cultivars BRS Pampa and IRGA 424 RI were used. During the 2020/21 harvest, sowing occurred in the second half of October using the cultivars BRS Pampa, BRS Pampeira, and IRGA 424 RI. In the first agricultural harvest, the climate characteristics for the region were El Niño (weak intensity), while the second harvest was characterized as a neutral year [24,25]. The maximum and minimum temperatures and solar radiation in the municipality of Capão do Leão throughout the crop cycle in the two growing seasons are shown in Figure 1.

The sowing density was 100 kg of seeds per hectare, and the soil presented the following physical and chemical characteristics: pH 5.2; calcium (2.3 cmolc dm^−3^); magnesium (1.7 cmolc dm^−3^); phosphorus (10.7 mg dm^−3^); potassium (33.0 mg dm^−3^); 1.9% organic matter, 18% clay; CTC with pH of 7 (7.3) and base saturation of 58%, being corrected according to technical recommendations for irrigated rice cultivation in southern Brazil [26]. Shading treatments were applied using shading-type screens with a shading of 35% at two moments during the reproductive period: upon reaching the phenological stage R0 and again from R4 to R9. The screens were installed 50 cm above the plant canopy. Control plants were maintained in environmental conditions without shading throughout the crop cycle. Pest and disease management, irrigation, fertilization, and harvesting were conducted according to technical recommendations for irrigated rice cultivation [27]. After installing the shade, plant tissues were collected at stages R2, R4, R6, and R8 for the analyses described below.

### 2.2. Leaf Temperature

The average temperature of the plants was monitored by measuring the temperature variation in the leaf surface using an FLIR infrared thermographic camera (FLIR E-5, FLIR Systems, Wilsonville, OR, USA). The model utilizes MSX technology, which enables the merging of images across the two bands of the electromagnetic spectrum (infrared and visible light), providing thermal images with enhanced definition while maintaining information quality. The thermographic camera was positioned 80 cm above the plant canopy, and images were captured on the collection days for destructive analysis at 10:00 a.m. Subsequently, the images were adjusted in FLIR Tools.

### 2.3. Quantification of Photosynthetic Pigments

Photosynthetic pigments were quantified according to the methodology proposed by Wellburn [28]. For this purpose, leaf disks from two young expanded leaves were used, weighing approximately 0.02 g of fresh sample, with four replications per treatment. The samples were read in a Molecular Devices spectrophotometer at absorbances of 480 nm, 649 nm, and 665 nm. The chlorophylls a and b and the total levels were calculated based on the following equations: chlorophyll *a* = (12.47 × A665) − (3.62 × A649); chlorophyll *b* = (25.06 × A649) − (6.5 × A665); total chlorophyll = chlorophyll *a* + chlorophyll *b*; and the results were expressed in mg g^−1^ of MF.

### 2.4. Specific Activity of Invertases

Extraction and incubation of acidic invertases from the vacuole (IAV), acidic from the wall (IAP), and neutral invertase from the cytosol (INC) were performed as described by Zeng [29] with adaptations. In total, 0.4 g of leaves macerated in liquid N_2_ were used, and 1.5 mL of extraction medium containing potassium phosphate buffer (200 mM, pH 7.5), phenylmethylsulfonyl fluoride (1 mM), and MgCl_2_ (5 mM) was added to each sample, as well as dithiothreitol (1 mM) and ascorbic acid (50 mM), for subsequent centrifugation at 18,000× *g* for 20 min at 4 °C.

The supernatant was collected for IAV and INC incubation, and the precipitate was collected for IAP incubation. In addition to the reagents used for IAV and INC, NaCl (1 M) and Triton-X-100 (1%) were also added for IAP extraction. The enzyme extract (500 μL) was added to 1000 μL of reaction medium containing 500 μL of sodium acetate buffer (pH 4.5 for IAV and IAP activity and pH 7.5 for INC activity), 200 mM of sucrose, and 5 mM of MgCl_2_. The reaction medium was incubated at 37 °C. After 10 and 40 min, 200 μL aliquots were collected to determine enzymatic activity through the quantification of reducing sugars produced, as described by the dinitrosalicylic acid (DNS) method of Miller [30], expressed in µmol of glucose g^−1^ min^−1^.

### 2.5. Quantification of Total Soluble Carbohydrates and Amino Acids

Approximately 250 mg of leaf tissue from two fully expanded leaves was used, with four replications per treatment. The material was macerated in 8 mL of M:C:W extracting solution (methanol/chloroform/water in a ratio of 12:5:3) and stored in amber bottles in the dark for 24 h. After this period, 2 mL of M:C:W solution was added, and the extract was centrifuged at 2500 rpm for 30 min. After centrifugation, 8 mL of the supernatant was transferred to Falcon tubes, and 2 mL of chloroform and 3 mL of ultra-pure water were added. Samples were centrifuged again for 30 min at 2500 rpm to facilitate phase separation. The upper phase was collected and concentrated by evaporation to approximately 50% of its original volume in a water bath at 30 °C to eliminate excess methanol and chloroform residues. The final extract was used for the quantification of total soluble sugars (AST) [31], sucrose [32], and total soluble amino acids (AASTs) [33].

The precipitate obtained in the first centrifugation, after drying at room temperature, was resuspended in 8 mL of 10% trichloroacetic acid (TCA). After 24 h at room temperature in the dark, 2 mL of 10% TCA was added, and the sample was centrifuged at 2500 rpm for 30 min. From the collected supernatant, water-soluble polysaccharides (PSAs) were quantified [31]. In total, 10 mL of 30% perchloric acid was added to the above precipitate. After shaking for 30 min, the tubes containing the reaction medium were centrifuged at 2500 rpm for 30 min. Starch was quantified from the collected supernatant [31].

AST and PSA quantification was carried out by adding the extracts, diluted in pure water, to tubes containing 1.5 mL of anthrone solution (0.15% in concentrated sulfuric acid) in an ice bath. After 15 min, the tubes were shaken and then incubated in a water bath at 90 °C for 20 min. They were subsequently kept in the dark until they reached room temperature.

Starch determination was carried out in the same manner as AST and PSA; however, at the end of the process, the values obtained were multiplied by a correction factor of 0.9 to convert them into starch content. To quantify sucrose, test tubes were bathed in ice, with extracts and 100 μL of 30% KOH added. After incubation at 100 °C for 10 min, when the sample reached room temperature, 3 mL of anthrone (0.15% in 70% sulfuric acid) was added, and the sample was incubated again at 40 °C for 15 min.

The total soluble amino acid contents were determined from 200 µL of extracts plus 0.5 mL of 0.2 M of citrate buffer with pH 5.0, 0.2 mL of reactive ninhydrin (5%) in ethylene glycol monomethyl ether, and 1 mL of KCN (2%) in ethylene glycol monomethyl ether (prepared from 0.01 M of KCN solution in pure water). The capped test tubes were incubated in a water bath at 100 °C for 20 min. After 20 min at room temperature, 1.3 mL of 60% ethanol was added.

Readings were taken on a UV-1900 UV-VIS Shimadzu spectrophotometer at wavelengths of 620 nm for total soluble sugars, starch, water-soluble polysaccharides, and sucrose, and 570 nm for total soluble amino acids.

### 2.6. Nutrient Content in Leaves

Fully expanded leaves from the middle third were collected and placed in an oven at 65 °C until they reached a constant weight and then crushed in a mill. Approximately 200 mg was weighed on an analytical balance for subsequent sulfuric digestion of macronutrients, and 500 mg for the nitro-perchloric digestion of micronutrients. Nitrogen (N) was subsequently read from the digested material (nitrogen distiller TE-0364) [34]; phosphorus (P)—UV spectrophotometer at 660 nm; potassium (K)—Flame Photometer (Micronal B462); calcium (Ca); magnesium (Mg); zinc (Zn); copper (Cu); manganese (Mn) and iron (Fe)—Flame Atomic Absorption Spectrophotometer (Model AA 990F—PG Instruments brand). To this end, the methodology proposed by Tedesco [35] was followed. The analyses described above were conducted in the Plant Nutrition and Fertilization Laboratory and the Chemistry Laboratory, both of which are part of the Postgraduate Program in Soil and Water Management and Conservation (MACSA) at the Federal University of Pelotas, RS, Brazil.

### 2.7. Productivity and Weight of a Thousand Grains

To evaluate grain productivity, manual harvesting was conducted in the valuable area of each plot (4.76 m^2^), with an average humidity of 22%. This material was subjected to threshing, weighing, and determination of grain harvest moisture, corrected to 13%, to estimate productivity.

In an area of 0.25 cm^2^, all panicles were collected, and from there, the number of whole and empty grains was determined. The sterility calculation was obtained by the relationship between the number of empty grains and the total number of grains, expressed as a percentage.

The weight of a thousand grains was determined according to the methodology described in the Seed Analysis Rules [36], using eight repetitions of 100 grains to estimate the weight.

### 2.8. Statistical Analysis

Data were collected in both agricultural years and analyzed separately. When the trend of results was similar, the average of the two years of cultivation was taken for the levels of photosynthetic pigments, invertases, nutrients, and carbohydrates. The experimental design employed was a randomized block design, with four replications in a 3 × 3 factorial scheme (cultivars × treatments). The data obtained were analyzed for normality using the Shapiro–Wilk test, taking into account the assumptions. Analysis of variance (ANOVA) was performed using Rbio software [37]. Subsequently, mean comparisons were conducted using Tukey’s test at a 5% significance level. Interaction effects between cultivars and treatments (cultivar × treatment) were incorporated into the ANOVA model and statistically evaluated. The significant interactions detected (*p* < 0.05) are analyzed and discussed in Section 3. In the absence of significant interactions, the main effects of cultivars and treatments were interpreted independently.

## 3. Results

### 3.1. Leaf Temperature

**Figure 2:**
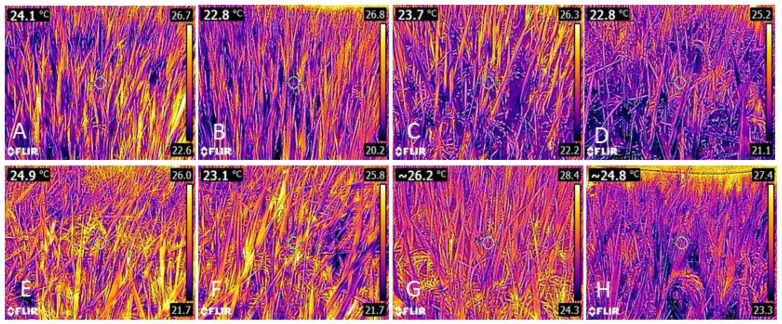
Thermographic images of rice leaves under the two growing conditions. (**A**) IRGA 424 RI control; (**B**) IRGA 424 RI shaded; (**C**) BRS PAMPA control; (**D**) BRS PAMPA shaded during the R4 phenological stage. (**E**) IRGA 424 RI control; (**F**) IRGA 424 RI shaded; (**G**) BRS PAMPA control; (**H**) BRS PAMPA shaded during the R8 phenological stage—year 2019/2020.

**Figure 3:**
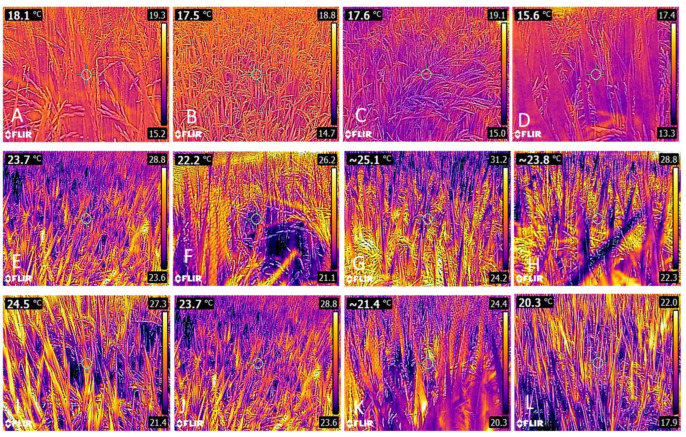
Thermographic images of rice leaves under the two growing conditions. (**A**) IRGA 424 RI control; (**B**) IRGA 424 RI shaded; (**C**) BRS PAMPA control; (**D**) BRS PAMPA shaded; (**E**) BRS PAMPEIRA control; (**F**) BRS PAMPEIRA shaded during the R4 phenological stage. (**G**) IRGA 424 RI control; (**H**) IRGA 424 RI shaded; (**I**) BRS PAMPA control; (**J**) BRS PAMPA shaded; (**K**) BRS PAMPEIRA control; (**L**) BRS PAMPEIRA shaded during the R8 phenological stage—year 2020/2021.

The results of leaf temperature assessments, as measured through thermographic images at stages R4 and R8, demonstrated that, regardless of the year and cultivar, leaf temperature was consistently lower in the shaded condition. On average, shading reduced to 1.5 °C for IRGA 424 RI and 1.1 °C for BRS Pampa in the first year of cultivation (Figure 2). In the second year, the average temperature reductions were 0.9 °C, 1.4 °C, and 1.3 °C for IRGA 424 RI, BRS Pampa, and BRS Pampeira (Figure 3).

### 3.2. Photosynthetic Pigments 

**Figure 4:**
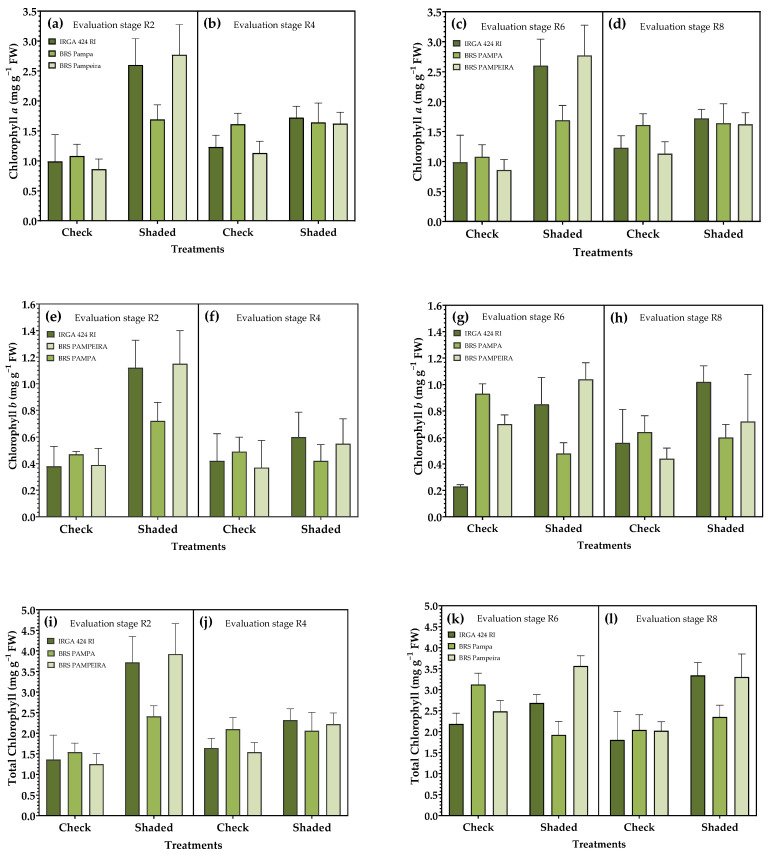
Effect of shading on photosynthetic pigments of irrigated rice cultivars. Chlorophyll *a* (**a**–**d**); chlorophyll *b* (**e**–**h**); total chlorophyll (**i**–**l**) in the reproductive period. R2, R4, R6, and R8 represent phenological stages evaluated within each of the solar radiation restriction periods: (R0–R4) and (R4–R9). Error bars correspond to the 95% confidence interval.

The chlorophyll *a* content differed between treatments in the four evaluation periods, with the highest values generally obtained in the shaded treatment R0–R4 (Figure 4a,b). When shading was applied during the R4–R9 period (Figure 4c,d), the same trend was observed; however, in the control condition, BRS Pampeira exhibited a higher chlorophyll a content when evaluated at the R6 stage. Among the cultivars in the control condition, at stages R4 and R6, cv. BRS PAMPA was superior to the others, whereas in the shaded condition, it presented the lowest values in stages R2, R6, and R8.

For chlorophyll *b*, there was a significant difference between treatments in all evaluation periods. Among the cultivars, IRGA 424 RI and BRS Pampeira exhibited the highest levels of this pigment in the shaded treatment across all evaluated periods (Figure 4e–h).

For the total chlorophyll content, shaded treatments presented the highest values in both restriction periods for the three cultivars, except in R6, where BRS PAMPA presented a higher total chlorophyll content in the control condition. Furthermore, for this same cultivar in R4 and R8, no significant differences were observed between treatments (Figure 4i–l).

### 3.3. Activity of Invertases

**Figure 5:**
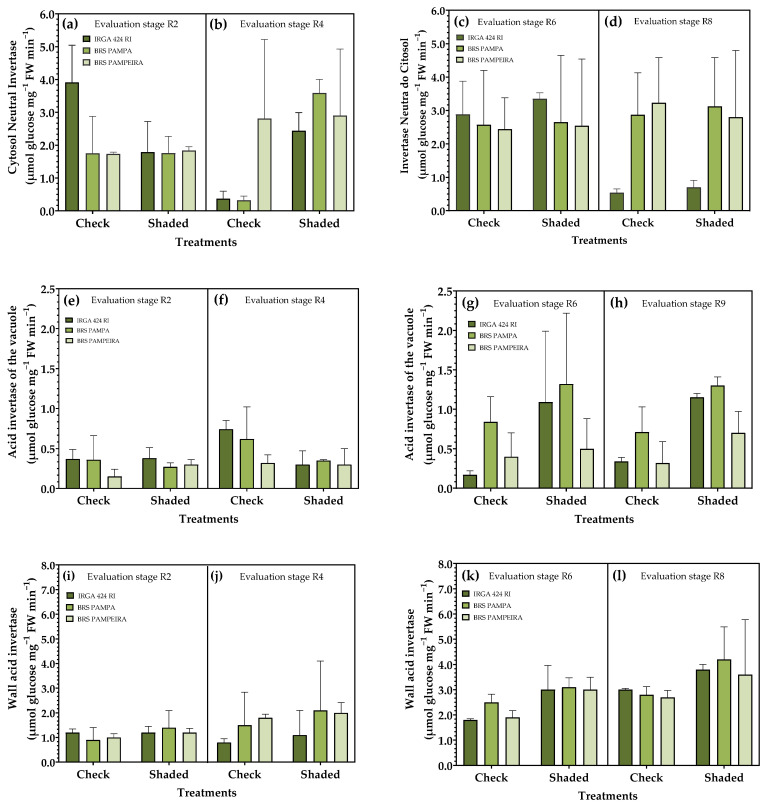
Effect of shading on enzymatic activity related to sucrose metabolism in leaves. Cytosol neutral invertase (**a**–**d**), acid invertase from the vacuole (**e**–**h**), and acid invertase from the cell wall (**i**–**l**) in the reproductive period. R2, R4, R6, and R8 represent phenological stages evaluated within each of the solar radiation restriction periods: (R0–R4) and (R4–R9). Error bars correspond to the 95% confidence interval.

An analysis of enzymatic activity related to sucrose metabolism (Figure 5a–d) showed a significant increase in cytosol neutral invertase (INC) activity in the shaded treatment in R4 for all cultivars analyzed. In the evaluation carried out in R2, for the cultivar IRGA 424 RI, the response is opposite, with greater enzyme activity in the control condition. In R8, only the cultivars differed, where the highest INC activity was observed in BRS Pampa and BRS PAMPEIRA (Figure 5d). Vacuole acid invertase (IAV) activity was higher in the control condition when evaluated in R4 in the cultivars IRGA 424 RI and BRS Pampa, while in R6, the opposite was observed: greater IAV activity for the cultivars IRGA 424 RI and BRS PAMPEIRA in the shaded condition, unlike R8 where all cultivars showed greater enzymatic activity in the shaded condition (Figure 5h).

Regarding cell wall acid invertase (IAP), a significant difference was observed between treatments, with the highest enzyme activity in the shaded condition. Regarding the cultivars, there was no response pattern at the R2 stage; cv. IRGA 424 RI showed greater activity in the control condition, and in R4, the response was the opposite, with lower enzyme activity in this cultivar for both treatments (Figure 5e).

### 3.4. Carbohydrate Metabolism in Leaves

**Figure 6:**
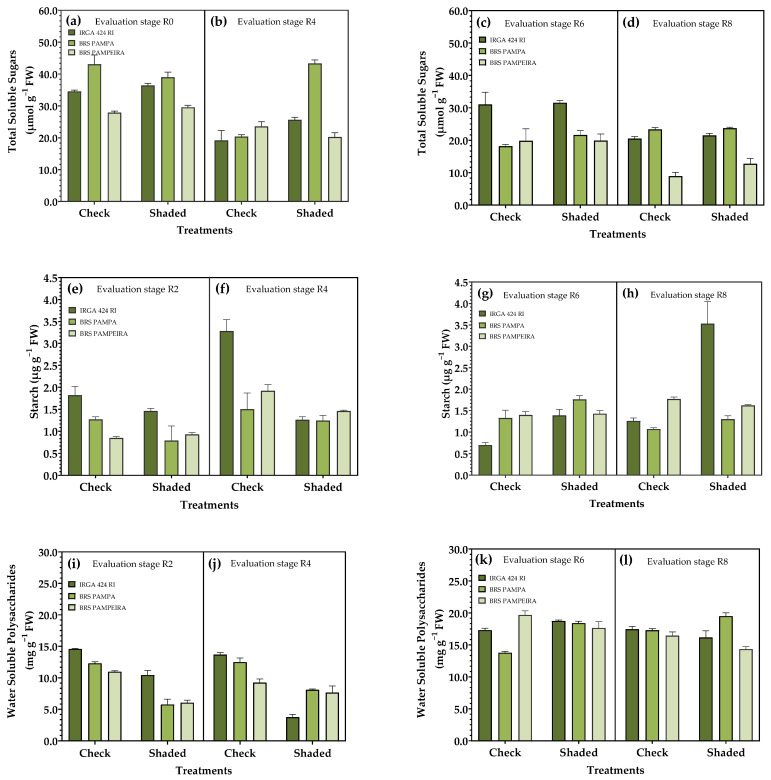
Effect of shading on carbohydrate metabolism of irrigated rice cultivars. Total soluble sugars (**a**–**d**), starch (**e**–**h**), and water-soluble polysaccharides (**i**–**l**) in the reproductive period. R2, R4, R6, and R8 represent phenological stages evaluated within each period of solar radiation restriction: (R0–R4) and (R4–R9). Error bars correspond to the 95% confidence interval.

During the first shading period (R0–R4), a significant difference was observed between the treatments in both evaluations. In periods R2 and R4, IRGA 424 RI presented the highest values of total soluble sugars (ASTs) in the shaded treatment, while BRS PAMPA differed only in the evaluation carried out in R4. This same cultivar presented the highest AST levels when shaded during the R0–R4 period (Figure 6a,b). When the restriction occurred during the R4–R9 period, BRS PAMPA showed the highest levels in the shaded treatment compared to the control, as evaluated in the R6 period. In contrast, in R8, only BRS PAMPEIRA exhibited this response (Figure 6c,d).

For starch content, in the first period of shading (R0–R4), the control treatment presented the highest values, and the same was observed for the cultivars IRGA 424 RI and RS PAMPA. When shading occurs in the period between R4 and R9, the opposite is observed: higher starch levels in the shaded treatment for these cultivars (Figure 6e–h).

Water-soluble polysaccharides (PSAs) differed across all evaluated periods, with the highest levels observed in the control treatment during the first restriction period for the three cultivars (Figure 6i–l). When shading occurred between R4–R9, IRGA 424 RI, and BRS PAMPA, the treatments showed opposite behavior in the evaluation in R6, with higher PSA levels for the shaded treatment (Figure 6k,l).

**Figure 7:**
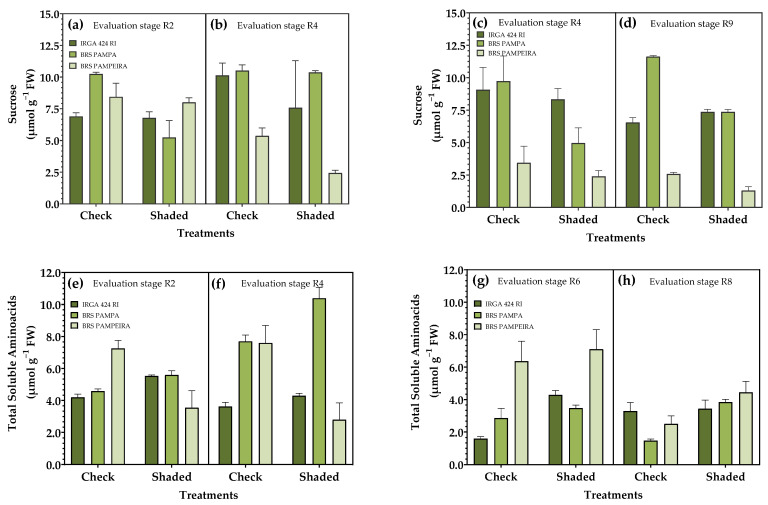
Effect of shading on carbohydrate metabolism of irrigated rice cultivars. Sucrose (**a**–**d**) and total soluble aminoacids (**e**–**h**) in the reproductive period. R2, R4, R6, and R8 represent phenological stages evaluated within each period of solar radiation restriction: (R0–R4) and (R4–R9). Error bars correspond to the 95% confidence interval.

The sucrose content, in general, was higher in the control treatment in both periods of shading. Compared to the other cultivars, BRS PAMPEIRA had the lowest sucrose levels in the second restriction period, regardless of the treatment (Figure 7a–d). Total soluble amino acids (AASTs) differed between treatments in all periods evaluated. For the cultivars IRGA 424 RI and BRS PAMPA, the shaded treatment resulted in a greater accumulation of AASTs in all evaluated periods. In contrast, for BRS PAMPEIRA, the opposite was observed; during the shading period (R0–R4), higher levels of AASTs were found in the control condition (Figure 7e–h).

### 3.5. Nutrient Content in Leaves

Shading generally reduced the nitrogen (N) and potassium (K) contents in the leaves. Regardless of the shading period, more significant N accumulation was observed in the control condition for the IRGA 424 RI and BRS PAMPA cultivars. Among cultivars, no pattern of accumulation was observed within treatments. In the control condition, when analyzed at the R2 and R4 stages, the cultivars mentioned above presented the highest levels compared to the BRS PAMPEIRA cultivar. When the evaluations took place in R6 and R8, the opposite was observed: higher N content for BRS PAMPA and BRS PAMPEIRA compared to IRGA 424 RI (Table 1).

For phosphorus (P) content, the response was the opposite; higher *p* values were found in the shaded condition in the first evaluation period. In R2 in the control condition, BRS PAMPA and BRS PAMPEIRA presented higher values than IRGA 424 RI, unlike the R4 period, where BRS PAMPEIRA was the cultivar with the least P accumulation in the leaves. In the second period, there was no pattern of behavior for treatments and cultivars (Table 1).

K accumulation in leaves was higher in the control condition in both evaluation periods for the three cultivars studied. Except for BRS PAMPEIRA in periods R2 and R6, K accumulation was more significant in the shading treatment (Table 1).

For the calcium (Ca) and magnesium (Mg) content in the leaves, significant differences were observed between treatments, with the highest values found in the shaded treatment in both periods of restriction. When shading occurred in R0–R4, IRGA 424 RI exhibited the lowest leaf Ca content among the other cultivars analyzed (Table 1).

Leaf copper (Cu) levels were higher in shaded treatments in both evaluation periods, stages R2 and R4, and R6 and R8 (Table 2). Among the cultivars, in the first period of shading (R0–R4), BRS PAMPEIRA presented the lowest Cu values compared to the other cultivars analyzed, regardless of the treatment (Table 2).

As for zinc (Zn), evaluated in the R2 and R6 stages, the shaded plants presented higher values than the control, unlike what was observed in the R4 and R8 periods. Analyzing the cultivars, IRGA 424 RI was superior to the others at stages R2 and R6, regardless of the treatment imposed (Table 2).

Iron (Fe) content was higher in the control treatment in both evaluation periods for the IRGA 424 RI and BRS PAMPA cultivars. In the BRS PAMPEIRA cultivar, the evaluations in R2 and R4 showed the opposite trend, with more significant Fe accumulation in the shaded condition (Table 2).

When the evaluations took place in periods R2 and R6, the manganese (Mn) content was higher in the control condition compared to the shaded condition. In contrast, in R8, the opposite occurred, with a more significant accumulation of Mn in the shaded treatment (Table 2). Regarding cultivars, in periods R2 and R6, IRGA 424 RI had the lowest Cu content compared to the others studied (Table 2).

#### Yield, Spikelet Sterility, and Weight of a Thousand Grains

The results presented in Table 3 show a statistically significant difference between the treatments (*p* ≤ 0.05). The yield and weight of one thousand grains were reduced by shading, and the cultivars BRS PAMPA and BRS PAMPEIRA were the most affected by this condition. On the other hand, the response is the opposite for spikelet sterility, with higher values for the shaded treatment at the R4–R9 stage.

### 3.6. Productivity, Spikelet Sterility, and a Thousand-Grain Weight

The results presented in the table below demonstrate a significant difference between the treatments (*p* ≤ 0.05). The productivity and weight of a thousand grains were reduced by shading, with the cultivars BRS PAMPA and BRS PAMPEIRA being the most affected by this condition. For spikelet sterility, the response is the opposite, with higher values for the shaded treatment at the R4–R9 stage (Table 3).

## 4. Discussion

Solar radiation is an essential environmental factor in the growth and development of plants. It influences not only the photosynthetic rate and carbohydrate accumulation but also the production of biomass [38], which can unbalance the production of assimilates and, therefore, has the potential to limit plant growth and stability [39]. In this context, chlorophylls *a* and *b* are the most critical photosynthetic pigments, involved in the absorption and transmission of solar energy during photosynthesis. Studies conducted on rice [40,41] and wheat [42] demonstrate that shading increases chlorophyll content in leaves, thereby enhancing light absorption capacity and improving usage efficiency to compensate for the effects of low light, which corroborates the results found in the present study (Figure 4). However, even if the chlorophyll content increases under shading conditions, the photosynthetic rate of the leaves is lower due to the lower availability of solar radiation and increased diffuse light [40]. Thus, a high total chlorophyll content in leaves under low-light conditions can be attributed to the increase in the number and size of chloroplasts, the amount of chlorophyll per chloroplast, and better grana development [43].

Consistent with the findings of the present study, Zivcak et al. [44] reported that shading, by reducing both the photosynthetic rate and the red/far-red light ratio, leads to a decrease in leaf sucrose content. In addition to serving as metabolic and structural resources for cells, soluble sugars also function as regulatory signals involved in various processes related to plant growth and development. The stress caused by shading conditions may have been responsible for altering the sucrose signaling pathway and carbon partitioning, as evidenced by the activity profile of specific invertase isoforms (Figure 5). Carbon partitioning under variable light conditions has been demonstrated in several studies. Sergeeva et al. [45] demonstrated that the inv4 mutant of *Arabidopsis thaliana*, which lacks vacuolar invertase activity, exhibited delayed initiation of photosynthesis and increased sugar accumulation, thereby disrupting the normal carbon flow from source to sink. Similarly, González et al. [46] found that vacuolar invertase activity in sugar beet petioles varied with the photoperiod and developmental stage, indicating a role in diurnal regulation of carbon allocation. Roitsch and González [47], as well as Koch [48], emphasized that invertase isoforms act not only as metabolic enzymes but also as signaling hubs, modulating carbon flow between source and sink tissues. Moreover, Weiszmann et al. [49] highlighted the importance of vacuolar invertase in stabilizing photosynthesis under stress conditions by preventing feedback inhibition due to sugar accumulation.

Shading of 35% during the reproductive phase of rice crops enables greater activity of IAP (cell wall acid invertase), which acts on the degradation of sucrose for use in sites of greatest need, thereby creating a concentration gradient between different carbohydrate unloading sites via the phloem (Figure 5). Sucrose is the primary form of translocation for photosynthetically assimilated sugars in plants. Its transport occurs via both apoplastic and symplastic routes. Symplastic transport does not require energy expenditure; it is a passive process via plasmodesmata; however, these are influenced by light intensity, where high-luminosity conditions result in a more accelerated development of plasmodesmata, unlike what is observed in shaded conditions. As a result, sucrose is slowly transported along the gradient, leading to feedback inhibition of photosynthesis [50]. The breakdown of sucrose by invertase enzymes makes carbon and energy available for respiration and compound synthesis [51]. Therefore, the greater IAP activity observed during the R2 period (Figure 5), when shaded, may be related to the higher P content in the leaves during the same period.

As for AST content, the response is opposite to sucrose in the shaded treatment, and higher levels were found in this condition (Figure 6) due to plant growth being relatively slow under adverse conditions, thus reducing the need for photosynthetic products, so that large amounts of structural sugars can be accumulated in leaves [52]. Under shading conditions, the amino acid content in the leaves was higher (Figure 7), which may be associated with decreased use, as the low-light condition limits growth. Furthermore, the study by Chen et al. [53] demonstrates that most enzymes controlling amino acid biosynthesis are downregulated in response to shading and the proteolysis of chloroplast proteins, leading to the accumulation of free amino acids.

Except for N, macronutrients increased in at least one of the shading periods (Table 1). N metabolism is an essential substrate for energy metabolism that determines crop yield and quality. The plant’s photosynthetic capacity is associated with the nitrogen (N) status in the leaves, which is reduced under conditions of lower solar radiation availability [52]. Greater availability of solar radiation not only increases the photosynthetic capacity of leaves but also increases carbon translocation to the roots [54]. The partitioned carbon serves as both an energy source and a nutritional signal, driving increased nutrient absorption to compensate for the low remobilization of nutrients in leaves under shady conditions.

Shading, in general, increased mineral nutrient concentrations in the leaves. In this condition, the temperature was lower (Figure 3 and Figure 4). Reductions in air temperature associated with shading allowed for improved thermal stress, which may have increased mineral nutrient absorption. Our results showed that shading tends to increase nutrient concentrations in leaves, which may be related to greater stomatal conductance, potentially increasing transpiration and nutrient absorption in shaded conditions [55]. Furthermore, the higher concentrations of Mg in the leaves are associated with a higher concentration of photosynthetic pigments in shaded leaves (Figure 4).

As the yield of rice crops is closely related to the carbohydrates produced during photosynthesis, using light energy is essential to obtain high yields. Although rice plants exhibit an increase in light capture capacity under low-light intensity by increasing chlorophyll content, this is accompanied by a decrease in the net photosynthetic rate, saturation irradiance, and electron transport rate, as well as a limited supply of electrons for photoreactive metabolism [40]. Therefore, under shading stress, the production of photoassimilates in rice is limited, which consequently limits plant growth and development, impacting yield (Table 3).

Several studies have demonstrated that shading experiments conducted at different stages of crop growth yield differential responses. Shading after grain filling has the most significant impact on formation, quality, and productivity [56,57,58,59]. These results align with those found in the present study, where an increase in the content of photosynthetic pigments was observed (Figure 5), accompanied by a reduction in the accumulation of sucrose and starch (Figure 7), which directly reflects productivity (Table 3).

Some studies have established that shading stress reduces crop grain yield [40,60]. When stress occurs between the booting stage and anthesis, it primarily reduces yield by decreasing drainage capacity. In contrast, shading after anthesis interrupts the progress of grain filling, resulting in a lower yield [61]. In the present study, when shading was applied between panicle differentiation (stage R1) and anthesis (stage R4), a reduction was observed in all the yield parameters evaluated (Table 3), with greater losses in productivity for BRS PAMPEIRA. When shading occurs after R4, the weight of a thousand grains is reduced, and spikelet sterility is increased in the three cultivars studied, which significantly reduces productivity. This finding aligns with the results of Deng et al. [56] and Mo et al. [60], who reported that shading after the R3 stage significantly reduces rice yield, primarily by reducing grain filling.

## 5. Conclusions

Shading significantly alters the physiological and biochemical metabolism of irrigated rice, reducing photosynthetic efficiency and photoassimilate production. Although rice plants show adaptive responses like increased chlorophyll and nutrient content, these do not fully offset the decline in net photosynthesis under low light. The most severe impact on grain yield occurs when shading happens after anthesis, reducing spikelet fertility and grain filling, with BRS PAMPEIRA being the most sensitive cultivar.

These findings are relevant in the context of climate variability, especially during El Niño events that increase cloud cover and rainfall, leading to reduced solar radiation during critical developmental stages. The results can guide breeding programs to develop cultivars with better shade tolerance and inform agronomic practices such as adjusting sowing dates and optimizing canopy structure.

This study was conducted over two growing seasons (2020 and 2021) in Pelotas, Rio Grande do Sul, Brazil, which presented distinct climatic conditions, first under a weak El Niño and second in a neutral year. While this provides insight into shading responses, the experiment’s geographic and environmental scope was limited. Further studies in diverse environments are needed to broaden the applicability of these results.

## Figures and Tables

**Table 1:** Macronutrient content in leaves of three irrigated rice cultivars subjected to shading during the reproductive period.

			Treatments					Treatments			
	Cultivar	Stage	Control	R0–R4	Av	CV (%)	Stage	Control	R4–R9	Av	CV (%)
N g kg^−1^	IRGA 424 RI	R2	36.8 Aa	25.2 Cb	31.0	3.5	R6	22.1 Ba	19.3 Bb	20.7	5.5
		R4	31.0 Aa	24.0 ABb	27.5		R8	17.8 Ba	14.2 Bb	16.0	
	BRS PAMPA	R2	33.8 Aa	29.0 Bb	31.4		R6	26.0 Aa	21.4 Ab	23.7	
		R4	32.5 Aa	19.6 Bb	26.0		R8	22.7 Aa	18.2 Bb	20.4	
	BRS PAMPEIRA	R2	30.4 Bb	28.8 Bb	29.6		R6	27.3 Aa	23.3 Ab	25.3	
		R4	24.2 Bb	27.4 Ab	25.8		R8	20.8 Aa	23.8 Aa	22.3	
		CV%	8.5				CV%	10.0			
P g kg^−1^	IRGA 424 RI	R2	1.8 Bb	3.0 Aa	2.4	4.7	R6	1.8 Bb	1.6 Ab	1.7	4.2
		R4	2.4 Ab	2.3 Aa	2.4		R8	0.9 Bb	1.5 ABa	1.2	
	BRS PAMPA	R2	2.7 Aa	2.7 Aa	2.7		R6	2.0 Aa	1.6 Ab	1.8	
		R4	2.3 Ab	2.3 Aa	2.3		R8	1.4 Aa	1.4 Ba	1.4	
	BRS PAMPEIRA	R2	3.4 Aa	3.0 Aa	3.2		R6	1.3 Cb	1.4 Aa	1.4	
		R4	0.9 Bb	1.5 Ba	1.2		R8	1.4 Aa	1.6 Aa	1.5	
		CV%	9.8				CV%	7.6			
K g kg^−1^	IRGA 424 RI	R2	37.9 Aa	31.8 Bb	34.9	5.2	R6	12.6 Aa	11.6 Ab	12.1	3.1
		R4	17.6 Ba	12.0 Bb	14.8		R8	13.4 Aa	7.2 Cb	10.3	
	BRS PAMPA	R2	33.3 Ba	26.3 Cb	29.8		R6	12.5 Aa	11.4 ABb	12.0	
		R4	21.2 Aa	13.3 Bb	17.2		R8	11.7 Ba	9.3 Bb	10.5	
	BRS PAMPEIRA	R2	31.6 Bb	37.6 Aa	34.6		R6	8.7 Bb	10.8 Ba	9.7	
		R4	11.4 Cb	13.2 Bb	12.3		R8	12.9 Aa	11.8 Ab	12.3	
		CV%	9.1				CV%	4.1			
Ca g kg^−1^	IRGA 424 RI	R2	3.8 Bb	4.2 Ba	4.0	4.3	R6	2.4 ABb	2.3 Bb	2.4	9.8
		R4	3.3 Bb	2.8 Bb	3.0		R8	2.6 Bb	3.5 Aa	3.1	
	BRS PAMPA	R2	4.9 Ab	5.3 Aa	5.1		R6	2.3 Bb	2.9 Aa	2.6	
		R4	3.6 Bb	3.2 Bb	3.4		R8	2.3 Bb	1.7 Bb	2.0	
	BRS PAMPEIRA	R2	4.9 Ab	5.3 Aa	5.1		R6	2.0 Bb	3.3 Aa	2.7	
		R4	3.3 Bb	3.2 Bb	3.2		R8	2.3 Bb	1.7 Bb	2.0	
		CV%	4.2				CV%	4.7			
Mg g kg^−1^	IRGA 424 RI	R2	1.9 Bb	2.4 Ba	2.1	2.8	R6	2.3 Ab	2.5 Ab	2.4	6.8
		R4	2.1 Bb	2.1 Bb	2.1		R8	1.4 Bb	2.3 Aa	1.8	
	BRS PAMPA	R2	2.6 Ab	3.2 Aa	2.9		R6	1.5 Bb	1.7 Bb	1.6	
		R4	1.7 Bb	2.6 Ba	2.2		R8	1.6 Bb	2.4 Aa	2.0	
	BRS PAMPEIRA	R2	2.6 Ab	3.2 Aa	2.9		R6	1.6 Bb	2.2 Aa	1.9	
		R4	2.5 Bb	3.1 Aa	2.8		R8	1.6 Bb	1.2 Bb	1.4	
		CV%	3.2				CV%	4.9			

Means followed by the same letters do not differ from each other by the Tukey test (*p* < 0.05). Capital letters compare cultivars, and lowercase letters compare treatments (control and shading) within each evaluation period. CV: coefficient of variation; Av: average.

**Table 2:** Macronutrient content in leaves of three irrigated rice cultivars submitted to shade during the reproductive period.

			Treatments					Treatments			
	Cultivar	Stage	Control	R0–R4	Av	CV (%)	Stage	Control	R4–R9	Av	CV (%)
Cu mg kg^−1^	IRGA 424 RI	R2	3.8 Ab	4.8 Aa	4.3	7.5	R6	3.5 Ab	4.0 Aa	3.7	7.1
		R4	4.7 Ab	4.4 Ab	4.4		R8	1.6 Bb	3.7 Ba	2.7	
	BRS PAMPA	R2	2.6 Bb	3.5 Ba	3.3		R6	1.6 Bb	2.5 Ba	2.2	
		R4	1.4 Cb	3.2 Ba	2.2		R8	2.4 Ab	4.5 Aa	3.4	
	BRS PAMPEIRA	R2	2.5 Cb	2.7 Ca	3.1		R6	1.9 Bb	3.3 Aa	2.6	
		R4	2.6 Bb	3.6 Ba	2.8		R8	2.6 Ab	4.8 Aa	3.7	
		CV%	6.2				CV%	6.2			
Zn mg kg^−1^	IRGA 424 RI	R2	15.0 Ab	15.7 Aa	15.4	3.6	R6	25.5 Ab	33.3 Aa	29.4	3.7
		R4	14.5 Ba	11.2 Cb	12.8		R8	24.3 Ba	13.5 Cb	18.9	
	BRS PAMPA	R2	17.7 Bb	14.2 Ba	15.9		R6	15.8 Bb	20.8 Ba	18.3	
		R4	17.3 Aa	15.7 Ab	16.5		R8	13.7 Cb	24.4 Aa	19.0	
	BRS PAMPEIRA	R2	12.6 Bb	14.4 Ba	13.5		R6	15.2 Bb	16.5 Cb	15.9	
		R4	13.1 Cb	13.8 Bb	13.5		R8	30.6 Aa	16.1 Bb	23.3	
		CV%	4.0				CV%	3.1			
Fe mg kg^−1^	IRGA 424 RI	R2	112.2 Aa	68.8 Bb	90.5	4.7	R6	82.7 Aa	37.7 Cb	60.2	2.3
		R4	84.7 Aa	56.1 Bb	70.4		R8	81.9 Aa	66.8 Ab	74.3	
	BRS PAMPA	R2	91.8 Ba	69.7 Bb	80.7		R6	52.3 Ba	46.6 Bb	49.5	
		R4	66.6 Ca	47.6 Cb	57.1		R8	67.6 Ba	48.5 Bb	58.1	
	BRS PAMPEIRA	R2	73.2 Cb	101.6 Aa	87.4		R6	84.7 Aa	60.3 Ab	72.5	
		R4	63.2 Bb	98.6 Aa	80.9		R8	56.1 Ca	35.2 Cb	45.7	
		CV%	3.2				CV%	3.06			
Mn mg kg^−1^	IRGA 424 RI	R2	274.4 Ca	207.7 Bb	241	1.2	R6	147.5 Ca	108.9 Cb	128.2	1.5
		R4	126.7 Ba	108.2 Bb	117.4		R8	277.1 ABa	276.1 Ba	276.6	
	BRS PAMPA	R2	360.4 Aa	303.1 Ab	331.8		R6	212.8 Ba	129.8 Bb	171.3	
		R4	118.4 Ba	104.6 Bb	111.5		R8	266.3 Bb	313.4 Aa	289.9	
	BRS PAMPEIRA	R2	354.5 Aa	329.7 Ab	342.1		R6	384.3 Aa	328.4 Ab	356.3	
		R4	326.5 Ab	365.5 Aa	346		R8	295.3 Ab	322.7 Aa	309.0	
		CV%	2.5				CV%	3.1			

Means followed by the same letters do not differ from each other using the Tukey test (*p* < 0.05). Capital letters compare cultivars, and lowercase letters compare treatments (control and shading) within each evaluation period. CV: coefficient of variation; Av: average.

**Table 3:** Grain productivity, spikelet sterility, and thousand-grain weight of the irrigated rice cultivars were studied as a function of shading periods in two agricultural years.

		Agricultural Year					
		2019/20			2020/21		
		Shading Season			Shading Season		
	Cultivate	Check	R0–R4	R4–R9	Check	R0–R4	R4–R9
Productivity (kg ha^−1^)	IRGA 424 RI	13.91 Aa	9.91 Ac	11.39 Ab	13.60 Aa	11.28 Ab	10.45 Ab
	BRS PAMPA	12.11 Ba	8.64 Bb	9.89 Bb	12.16 Ba	9.95 Bb	7.89 Bb
	BRS PAMPEIRA	-	-	-	9.76 Ca	6.68 Cb	7.81 Bb
	Média	12.70	9.27	10.64	11.86	9.30	8.71
	CV (%)	7.66			8.0		
Spikelet sterility (%)	IRGA 424 RI	14.26 Ab	16.01 Ab	22.18 Aa	13.81 Bb	16.70 Bb	24.02 Ba
	BRS PAMPA	13.41 Ab	18.04 Aa	17.14 Ba	12.04 Bb	14.54 Bb	24.69 Ba
	BRS PAMPEIRA	-	-	-	24.70 Ab	37.57 Aa	36.03 Aa
	Média	13.84	17.03	19.66	14.81	20.38	25.92
	CV (%)	6.97			8.93		
Thousand-grain weight (g)	IRGA 424 RI	25.55 Aa	24.94 Ab	24.45 Ab	25.05 Aa	23.59 Ac	24.49 Ab
	BRS PAMPA	25.35 Aa	24.45 Ab	24.46 Ab	25.06 Ba	24.40 Bb	24.18 Bb
	BRS PAMPEIRA	-	-	-	25.72 Ba	24.14 Cb	24.20 ABb
	Média	25.45	24.7	24.46	25.27	24.04	24.29
	CV (%)	2.44			1.08		

Means followed by the same letters do not differ from each other using the Tukey test (*p* < 0.05). Capital letters compare cultivars, and lowercase letters compare treatments (control and shading) within each evaluation period. CV: coefficient of variation.

## Data Availability

The original contributions presented in this study are included in the article. Further inquiries can be directed to the corresponding author.
